# Nanomechanical and Material Properties of Fluorine-Doped Tin Oxide Thin Films Prepared by Ultrasonic Spray Pyrolysis: Effects of F-Doping

**DOI:** 10.3390/ma12101665

**Published:** 2019-05-22

**Authors:** Le Thi Cam Tuyen, Sheng-Rui Jian, Nguyen Thanh Tien, Phuoc Huu Le

**Affiliations:** 1 Ceramics and Biomaterials Research Group, Advanced Institute of Materials Science, Ton Duc Thang University, Ho Chi Minh City 700000, Vietnam; 2 Faculty of Applied Sciences, Ton Duc Thang University, Ho Chi Minh City 700000, Vietnam; 3 Department of Materials Science and Engineering, I-Shou University, Kaohsiung 840, Taiwan; 4 College of Natural Science, Can Tho University, 3-2 Road, Can Tho City 94000, Vietnam; nttien@ctu.edu.vn; 5 Department of Physics and Biophysics, Faculty of Basic Sciences, Can Tho University of Medicine and Pharmacy, 179 Nguyen Van Cu Street, Can Tho City 94000, Vietnam

**Keywords:** fluorine-doped tin oxide (FTO), nanoindentation, F/Sn atomic ratios, ultrasonic spray pyrolysis (USP)

## Abstract

Fluorine-doped tin oxide (FTO) thin films were deposited on glass substrates using ultrasonic spray pyrolysis (USP) at a fixed substrate temperature of 400 °C and various Fluorine/Tin (F/Sn) atomic ratios of 0, 0.1, 0.5, and 1.0. Effects of F/Sn atomic ratios on structural-morphological, compositional, electrical, optical, and nanomechanical properties of the FTO thin films were systematically studied. The FTO films exhibited a tetragonal structure with preferred orientations of (110), (200), and (211), and polycrystalline morphology with spear-like or coconut shell-like particles on the surfaces. The presence of F-doping was confirmed by XPS results with clear F1s peaks, and F-concentration was determined to be 0.7% for F/Sn = 0.1 and 5.1% for F/Sn = 0.5. Moreover, the resistivity of FTO films reduced remarkably from 4.1 mΩcm at F/Sn = 0 to 0.7 mΩcm at F/Sn = 1, primarily due to the corresponding increase of carrier concentration from 2 × 10^20^ cm^−3^ to 1.2 × 10^21^ cm^−3^. The average optical transmittance of the films prepared at F/Sn of 0–0.5 was over 90%, and it decreased to 84.4% for the film prepared at F/Sn = 1. The hardness (*H*) and Young’s modulus (*E*) of the FTO films increased when the F/Sn ratios increased from 0 to 0.5, reaching maximum values of *H* = 12.3 ± 0.4 GPa, *E* = 131.7 ± 8.0 GPa at F/Sn = 0.5. Meanwhile, the *H* and *E* reduced considerably when the F/Sn ratio further increased to 1.0, following the inverse Hall-Petch effect approximately, suggesting that the grain boundary effect played a primary role in manipulating the nanomechanical properties of the FTO films. Furthermore, favorable mechanical properties with large *H/E_f_* and $H^{3}/E_{f}^{2}$ ratios were found for the FTO film prepared at F/Sn = 0.5, which possessed high crystallinity, large grain size, and compact morphology.

## 1. Introduction 

Florine-doped tin oxide (FTO) thin films have attracted considerable attention because of their high electronic conduction and optical transparency in the visible region for technological applications, such as flat displays, thin-film solar cells, sensors, organic light emitting diodes, transparent heaters, and architectural glass [1,2,3,4]. FTO has attracted great interest because of its wide energy gap (*E_g_* = ~3.6 eV), low cost of production, thermal stability, chemical inertness, and high transparency [1,4,5,6,7]. However, the electrical conductivity of FTO remains lower than the ∼10^4^ S/cm of tin-doped indium oxide (ITO) [8]. Generally, the electrical properties of SnO_2_ films depend on the sizes of grains [9], and doping with Ta [10], Sb [11], or F [12,13,14]. Fluorine is a preferred dopant to improve the electrical conductivity of SnO_2_ films and retain the crystal structure because of the similar ionic radius between F^−^ (1.33 Å) and O^2^⁻ (1.32 Å). Fluorine atoms replace the oxygen sites in the lattice and donate free electrons to enhance the electrical conductivity of FTO samples [7].

FTO thin films have been extensively prepared by various methods, such as atmosphere pressure chemical vapor deposition (APCVD) [3,15,16], sputtering [4], sol-gel [7,12], and spray pyrolysis deposition (SPD) [1,13,17,18,19]. Among the various techniques, spray pyrolysis deposition (SPD) is a simple, economical, and commonly used method to prepare self-textured FTO thin films with a simple scalable deposition and easy doping process that is achieved by manipulating the substrate temperature, calcinations, gas pressure, and flow rate [1]. 

Besides the structural-morphological, electrical, and optical properties, the nanomechanical properties of TCO films are important parameters that determine the performance of devices involving TCO films. It is essential for designing stress-free thin-film, semi-transparent multi-layer structures and top-emitting organic light-emitting displays that involve one or more layers of TCO films on both rigid and flexible substrates [2]. The Young’s modulus and hardness are of the greatest interest, because they reflect the elastic deformation and resistance to permanent deformation. Nanoindentation is regarded as a good method to study the mechanical property of materials at the nanoscale level, including various nanostructures [20,21] and thin films [22,23,24,25,26,27,28,29,30,31,32]. G. Han et al. reported the enhanced nanomechanical properties of FTO thin films through the modification of the structure-morphology via the post-annealing process [15]. However, to the best of our knowledge, the F-doping- dependent nanomechanical properties of FTO films prepared by USP have not been studied yet.

In this study, we successfully employed ultrasonic spray pyrolysis (USP, a modified SPD technique) to fabricate FTO thin films on glass substrates. Especially, the atomizer was an ultrasonic nebulizer, which was originally used for treatment of respiratory disease (particularly asthma) using high-frequency vibrations to turn liquid medication into a mist. The structural, morphological, compositional, electrical, optical, and nanomechanical properties of polycrystalline FTO thin films prepared at F/Sn atomic ratios of 0, 0.1, 0.5, and 1.0 were systematically studied. The results in this study will provide a strategy for fabricating high-quality FTO thin films with the enhanced both desired material and nanomechanical properties for applications.

## 2. Experimental Details

**Figure 1:**
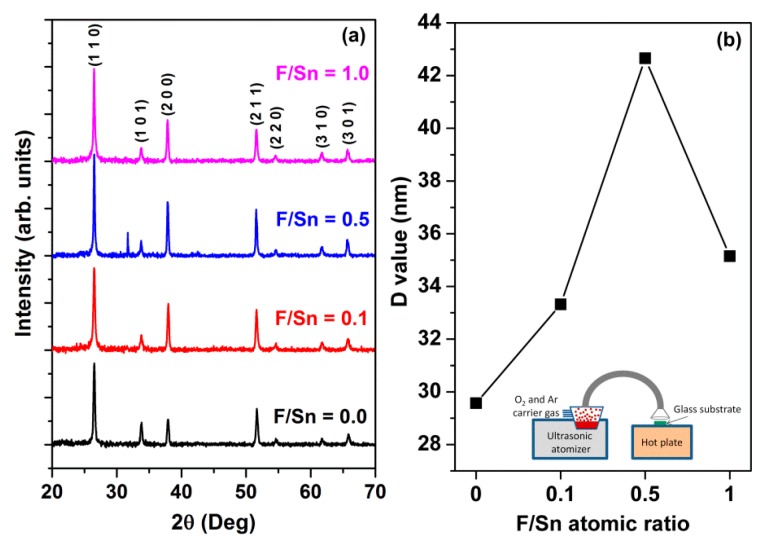
(**a**) X-ray diffraction (XRD) patterns and (**b**) grain size (*D*) of the fluorine-doped tin oxide (FTO) thin films prepared at various F/Sn ratios of 0, 0.1, 0.5, and 1. The inset in (**b**) is a schematic of the ultrasonic spray pyrolysis (USP) system.

Florine-doped SnO_2_ thin films were deposited on Corning glass substrates (15 × 15 × 1 mm^3^) at a substrate temperature of 400 °C and a mixed oxygen-argon carrier gas at a flow rate of 1.5 L/min, and at various F/Sn atomic ratios of 0, 0.1, 0.5, and 1.0 using USP. In a typical procedure, 5.64 g SnCl_2_·2H_2_O was dissolved in 25 mL distilled water. To improve the solubility of the solution, 2 mL of HCl was added to the solution and stirred vigorously (~30 min) until the solution turned transparent. Then, NH_4_F with appropriate weights were added to the solution to introduce different F-doping levels at F/Sn = 0, 0.25, 0.5, and 1. The solution was then stirred at 600 rpm for approximately 30 min until the solution turned transparent. The product solution was placed in an ultrasonic nebulizer reactor, which can produce an aerosol with a controlled droplet size around 0.5–5 μm depending on the precursor solution. The experimental apparatus consists of a specific homemade ultrasonic atomizer, spray gun, and graphite hotplate (see Figure 1b inset). The ultrasonic atomizer consisted of medical equipment with a piezoelectricity ultrasonic transducer inside, providing a frequency of 1.6 MHz ± 5%. Two gas pipes beyond the precursor container were made for conducting oxygen and argon carrier gases (Figure 1b inset). The deposition time was 7 min, and the film thickness was in range of 286–317 nm.

The crystal structure of FTO thin films was determined through X-ray diffraction (XRD; Bruker D2) using CuK*_α_* radiation (*λ* = 1.5406 Å) in 2*θ*-*θ* configuration. Surface morphology and film thickness were examined using field-emission scanning electron microscopy (SEM, JEOL JSM-6500, Pleasanton, CA, USA) through plane-view and cross-sectional images, respectively. The surface feature and roughness of FTO thin films were characterized through atomic force microscopy (AFM; Topometrix-Accures-II, Topometrix Corporation, Santa Clara, CA, USA). The center line average (*R_a_*) was used to present surface roughness. Detailed structural information at atomic scale of a selected FTO film was obtained from high-resolution scanning transmission electron microscope (HRTEM) images (JEOL JEM-ARM200F, Tokyo, Japan) operated at 200 kV. The surface chemical composition and F-doping concentration of FTO thin films were characterized using X-ray photoelectron spectroscopy (XPS; ThermoVG 350, East Grinstead, UK) with the X-ray source (MgK*_α_* 1253.6 eV, 300 W). The binding energies obtained in the XPS analysis were standardized using C1s at 285.0 eV. XPS curve fitting was performed using the freeware XPSPEAK 4.1 with the Shirley background subtraction, and assuming a Gaussian-Lorentzian peak shape. In-plane electrical conductivity, carrier concentration (*n*) and mobility (*μ*) were measured at room temperature by using a Hall system (Bio-Rad HL5500PC, Hercules, CA, USA) with van der Pauw geometry.

Nanomechanical properties (i.e., hardness and Young’s modulus) of the FTO thin films were obtained by nanoindentation tests (MTS NanoXP^®^ system, MTS Cooperation, Nano Instruments Innovation Center, Oak Ridge, TN, USA). Nanoindentation measurements were made employing a triangular pyramid Berkovich diamond indenter with curvature radius of 50 nm. The continuous stiffness measurement technique was used in the nanoindentation tests [33], which was performed by superimposing a small oscillation on the primary loading signal and analyzing the system response by using a lock-in amplifier. The indenter was loaded and unloaded three times to ensure that the tip was properly in contact with the film surface, and that any parasitic phenomenon was released from the measurements. Then, the indenter was loaded for the fourth and final time at a strain rate of 0.05 s^−1^ until an indent depth of 80–88 nm was achieved and held for 5 s at the peak load. Finally, the indenter was withdrawn with the same strain rate until 10% of the peak load was reached. At least 20 indents were performed on each sample. Each indentation was separated by 50 μm to avoid possible interferences between neighboring indents. The analytical method proposed by Oliver and Pharr was used to determine the hardness and Young’s modulus of the FTO thin films [34].

## 3. Results and Discussion

Figure 1a shows XRD patterns of the FTO films prepared at different F/Sn atomic ratios of 0, 0.1, 0.5, and 1.0. Clearly, the films exhibited tetragonal rutile structure of SnO_2_ with preferred orientations of (110), (200), and (211). The grain sizes (*D*) of the films were estimated by using the Scherrer equation *D* = 0.9*λ/β*cos*θ*, where *λ*, *β*, and *θ* are the X-ray wavelength, full width at half maximum of the FTO (110)- oriented peak, and Bragg diffraction angle, respectively. The estimated *D* values of FTO thin films increased from 29.6 nm at F/Sn = 0 to 42.7 nm at F/Sn = 0.5, and then decreased to 35.1 nm at F/Sn = 1, as shown in Figure 1b. The increased grain size with F-doping tendency was consistent with the result in Ref. [14], where the grain size increased from 20.5 nm for the pristine SnO_2_ film to 27.1 nm for the 12 wt.% F-doped SnO_2_ film. Noticeably, there is a small peak at ~31.7° for the film prepared at F/Sn = 0.5 that suggests the presence of Sn_3_O_4_ impurity phase in the film [35]. This is reasonable, as the Sn_3_O_4_ impurity phase is also detected in the FTO films prepared by USP [19]. 

**Figure 2:**
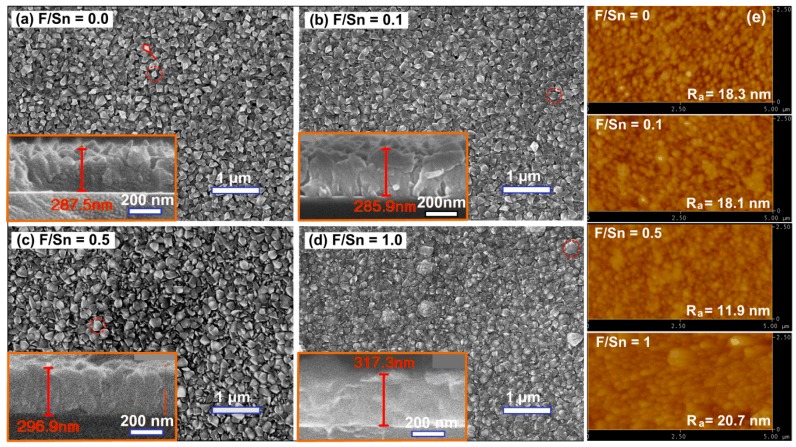
(**a**–**d**) Top-view and cross-section scanning electron microscopy (SEM) images of the FTO thin films prepared at F/Sn atomic ratios of 0, 0.1, 0.5, and 1.0; the dotted circles marked typical grain shapes; the film thicknesses are also written the cross-section SEM images. (**e**) Atomic force microscopy (AFM) images of the FTO films with the corresponding surface roughness values (*R_a_*).

Figure 2 presents the top-view and cross-section SEM images of FTO thin films deposited at 400 °C and various F/Sn ratios from 0 to 1. Clearly, all the films exhibited granular polycrystalline morphology with spear-like particles for F/Sn = 0 and coconut shell-like particles for F/Sn = 0.1, 0.5, and 1.0. Similar to the *D* result estimated by XRD, the particle sizes observed from SEM images appeared to be increased for F/Sn in 0–0.5 range and decreased for F/Sn = 1.0. Noticeably, some large clusters composed of smaller particles were found on the surface of the highest doping level film (i.e., F/Sn = 1). It is worthy of note that a particle size (observed from SEM images) can be larger than the grain (crystallite) size (obtained from XRD result), as it may consist of number of grains and even include amorphous regions. As shown in the insets of Figure 2a–d, the FTO films had the thickness in range of 285.9–317.3 nm and exhibited compact-uniform structure. Like the SEM results, AFM images show granular surface features with roughness (*R_a_*) of 11.9–20.7 nm (Figure 2e). The film grown at F/Sn = 0.5 achieved the lowest *R_a_* of 11.9 nm, suggesting that this doping level was one of the good conditions to prepare FTO films. 

**Figure 3:**
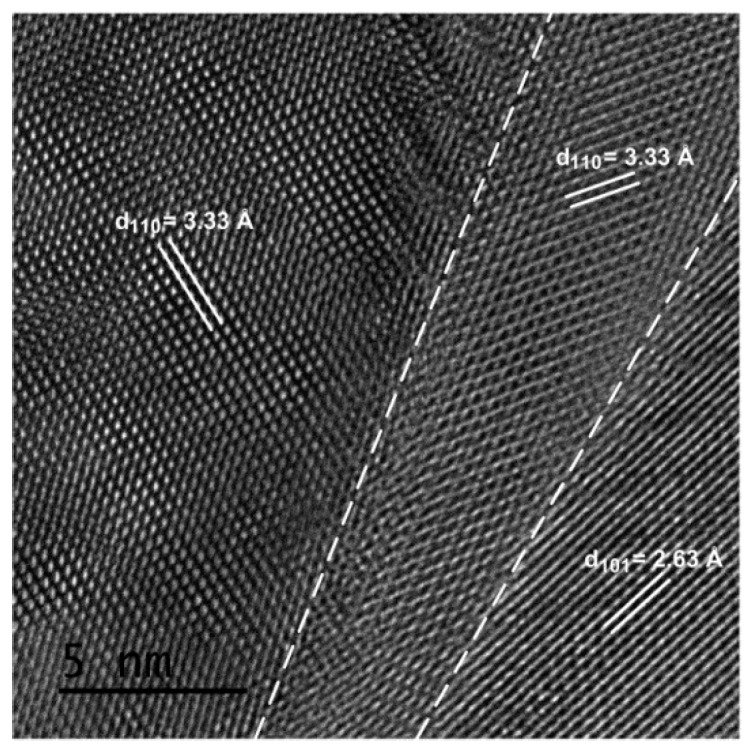
High-resolution scanning transmission electron microscope (HRTEM) image of the FTO film prepared at F/Sn = 0.5. Three domain crystallites are separated by the dashed lines.

To further understand the structural quality and grain-boundary structure of the FTO films, HRTEM was performed on the FTO film prepared at F/Sn = 0.5, and the result is shown in Figure 3. Clearly, the HRTEM image shows the boundary between three crystallites with sharp interfaces and high crystallinity. The lattice fringes have d-spacing of 3.33 Å and 2.63 Å, corresponding to (110) and (101) crystal planes of the rutile-type SnO_2_ structure. This result demonstrates that the FTO films in this study are compact and have good continuity. 

**Figure 4:**
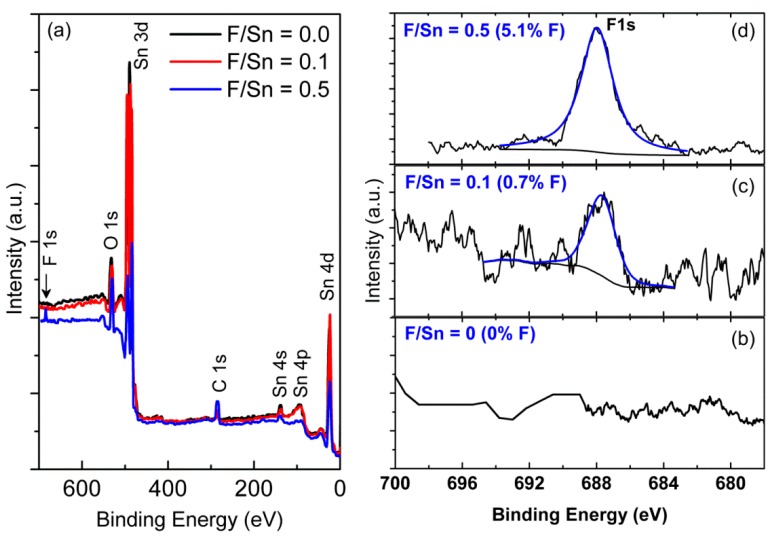
(**a**) XPS survey and F1s spectra of the surface layer of FTO films prepared at various F/Sn atomic ratios of 0, 0.1, and 0.5. (**b**–**d**) Core level spectra of F1s of the FTO films prepared at F/Sn of 0, 0.1, and 0.5.

Figure 4 shows the XPS survey and F1s spectra of FTO thin films prepared at 0, 0.1, and 0.5, in which the survey spectra were calibrated by the binding energy of C1s peak at 285.0 eV. Clearly, the main peaks of Sn3d, Sn4d, and O1s core levels are well pronounced, and small peaks of F1s were also observed for F/Sn = 0.1, 0.5, indicating the high purity of pristine and F-doped SnO_2_ thin films. Quantitative XPS analysis found that F-doping concentration increased when F/Sn atomic ratios increased, namely, F- concentration of 0, 0.7, and 5.1% for F/Sn of 0, 0.1, and 0.5, respectively (Figure 4b–d). The F-content of 5.1% was comparable to that of the FTO films (~5.0%) prepared by APCVD [3]. 

**Figure 5:**
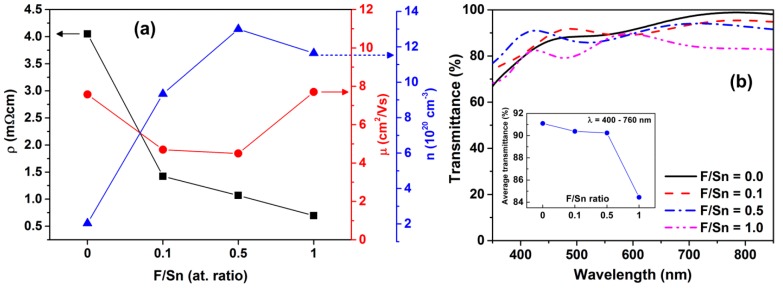
(**a**) F/Sn ratio-dependent resistivity (*ρ*, black squares), carrier concentration (*n*, blue triangulars), and carrier mobility (*µ*, red spheres) of the FTO thin films. (**b**) Transmission spectra of FTO thin films as a function of F/Sn ratios. The inset in (**b**) presents the average optical transmittance (%) of the films in the visible range of 400–760 nm.

The electrical properties (resistivity (*ρ*), carrier mobility (*μ*), and carrier concentration (*n*)) of the FTO thin films grown at various F/Sn atomic ratios are shown in Figure 5a. The *n* increased substantially from 2 × 10^20^ to 13 × 10^20^ cm^−3^ as F/Sn increased from 0 to 0.5, and then it decreased slightly to 11.6 × 10^20^ cm^−3^ at F/Sn = 1.0. When F⁻ anion substitutes an O^2-^ anion, free electrons will be generated for charge compensation to result in the increased *n* with increasing F/Sn from 0 to 0.5. However, there is a certain saturation limit that excess F⁻ cannot substitute O^2^⁻ anymore, and the F⁻ anions may be expelled to segregate on grain boundaries. This results in the slight decrease of *n* at F/Sn = 1. The present behavior of *n* vs. F-doping is consistent with those of the FTO films prepared by magnetron sputtering [36] and spray pyrolysis technique [14,37]. In contrast to the *n* variation, the *μ* decreased slightly from 7.6 to 4.5 cm^2^/Vs when F/Sn increased from 0 to 0.5, and then it increased to 7.7 cm^2^/Vs at F/Sn = 1. 

The mobility (*μ*) is described by Matthiessen’s rule [38], as follows:

(1) $$\frac{1}{\mu }=\frac{1}{\mu_{impurity}}+\frac{1}{\mu_{gb}}+\frac{1}{\mu_{phonon}}+\frac{1}{\mu_{hopping}}+\ldots$$

where *μ_impurity_*, *μ_gb_*, *μ_phonon_*, and *μ_hopping_* are factors that influence mobility from impurity scattering, grain boundary scattering, phonon scattering, and retardation by hopping transport, respectively. In TCO thin films, the ionized impurities and grain boundaries are found to be the main scattering mechanisms [13,38]. The electron mean free path (*L*) of thin films can be estimated by the equation $L={\left(3\pi^{2}\right)}^{1/3}\hbar e^{-1}\mu n^{1/3}$, where ℏ is the reduced Planck’s constant [13,38]. The *L* values of the present FTO films are in range of 0.9–1.7 nm, which is far smaller than grain size; thus, grain boundary scattering is not the dominant scattering mechanism. A higher *n* usually creates more scattering centers that lead to reduced *μ*, as shown in Figure 5a. Due to the aforementioned results of *n* and *μ*, the resistivity monotonically decreased from 4.1 to 0.7 mΩcm with increasing F/Sn from 0 to 1, as 1/*ρ* = *nμ*|*e*|, where *e* is the electron charge. This F-doping-dependent *ρ* is consistent with that for the FTO films [13]. 

As shown in Figure 5b, the average optical transmittances in the visible range (*λ* = 400–760 nm) of the 286–317 nm-thick films were 91.1, 90.4, 90.2, and 84.4% for F/Sn ratios of 0, 0.1, 0.5, and 1, respectively. This means that the average transmittance remains above 90% for F/Sn ≤ 0.5, which is higher than the value of 80% for the FTO prepared by APCVD [3], and comparable with that of the relevant FTO films prepared by the sol-gel process [12]. Moreover, the transmittance of the films prepared at the highest F-doping level (F/Sn = 1) reduces considerably to 84.4%, which could be due to the scattering effect due to the increase of surface roughness to *R_a_* = 20.7 nm and defect concentration. The present F-doping-dependent transmittance is consistent with the result for the FTO films prepared by the sol-gel process [12]. 

Table 1. summarizes the electrical and optical properties of the FTO films in this study in comparison with those of the relevant or optimal FTO films in the literature [3,13,17,18,19]. The lowest *ρ* of 0.7 mΩcm for the film prepared at F/Sn = 1.0 in this study is considerably higher than those of the FTO films with *ρ* in range of 1.5–6.5 mΩcm [13,18,19], but was slightly lower than the *ρ* values of the FTO films [17,18]. In additions, the transmittance at wavelength of 550 nm of the present films was comparable or higher than that of the FTO films in Refs. [3,13,17].

**Figure 6:**
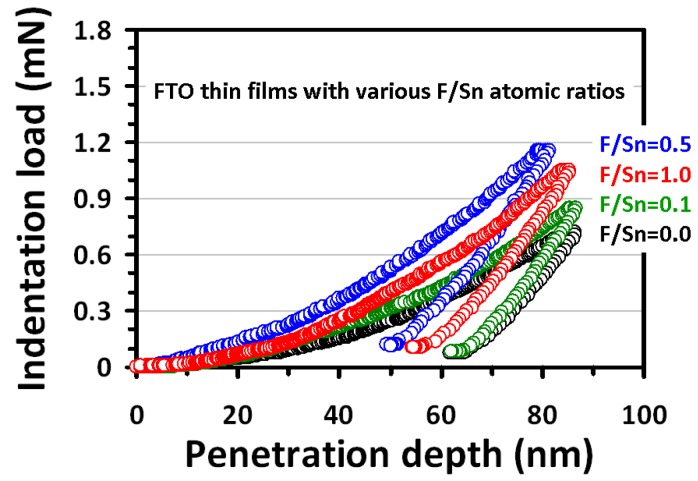
Load-displacement (*P–h*) curves of the FTO thin films prepared at F/Sn of 0, 0.1, 0.5, and 1.0.

All nanoindentation tests were performed at an indentation depth of approximately 30% film thickness to avoid surface and substrate effects [39]. Figure 6 shows the typical load-displacement (*P*–*h*) curves of FTO thin films prepared at different F/Sn ratios from 0 to 1, which provides the information about elastic behavior and plastic deformation. From the *P*–*h* data, we employed the Oliver and Pharr method to calculate the hardness (*H*) and Young’s modulus ($E_{f}$) of the thin films [34]. 

Particularly, the hardness was estimated as *H = P_m_/A_p_*, where *P_m_* and *A_p_* are the maximal indentation load and the projected contact area of indentation, respectively. The *A_p_* was determined from indenter tip calibration and is a function of contact depth (*h_c_*), namely, $A_{p}=24.56h_{c}^{2}$ for a perfectly sharp Berkovich [33]. The contact stiffness (*S*) is determined as *S* = *dP*/*dh*, i.e., the slope of the initial portion of the unloading curve. The contact depth can be estimated from the *P–h* data using $h_{c}=h_{m}-\varepsilon (P_{m}/S)$, where *ε* is the indenter constant (0.75 for a Berkovich indenter tip) and $h_{m}$ is the maximum indentation depth. The elastic modulus of materials is calculated using the Sneddon relation [40] $S=2\beta E_{r}\sqrt{A_{p}/\pi }$, wherein *β* is a geometric constant (*β* ≈ 1 for a Berkovich indenter tip). The reduced elastic modulus (*E_r_*) is determined as follows:

(2) $$\frac{1}{E_{r}}=\frac{\left(1-v_{f}^{2}\right)}{E_{f}}+\frac{\left(1-v_{i}^{2}\right)}{E_{i}}$$

Here, *v* is the Poisson’s ratio and *i* and *f* denote parameters for the indenter and FTO thin films, respectively. The elastic modulus ($E_{i}$) and Poisson ratio ($\upsilon_{i}$) of the Berkovich indenter used in this study were 1141 GPa and 0.07, respectively [34]. The $\upsilon_{f}$ was assumed to be 0.25 by referencing the Poisson’s ratio of thin films [29,41,42].

**Figure 7:**
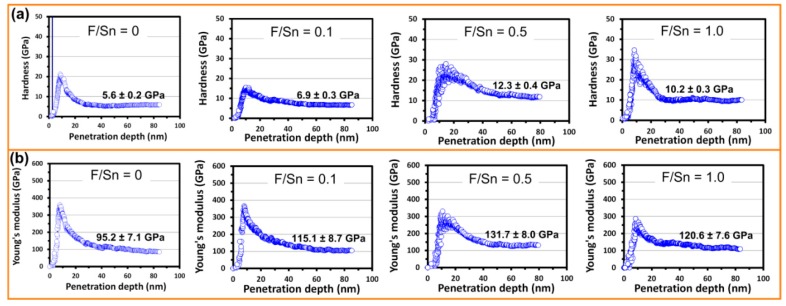
(**a**) Hardness and (**b**) Young’s modulus of the FTO thin films prepared at F/Sn of 0, 0.1, 0.5, and 1.0.

Figure 7a,b shows the calculated hardness-displacement (*H*–*h*) and Young’s modulus–displacement (*E*–*h*) curves of the films prepared at different F/Sn ratios. The *H*–*h* and *E*–*h* curves can be divided into two stages, i.e., (1) the *H* or *E* gradually increases to reach a maximum value as *h* is increased and (2) subsequently decreases to an almost constant value. The first stage is usually attributed to the transition from purely elastic to elastic/plastic contact, and the hardness is not accurately measured by the mean contact pressure at this stage. Instead, the mean contact pressure can represent the hardness only under the condition of a fully developed plastic zone. After the first stage, the *H* and *E* reach the constant values, which are regarded as intrinsic properties of the films. As shown in Figure 7a,b, the hardness of the FTO films deposited at F/Sn = 0, 0.1, 0.5, and 1.0 was 5.6 ± 0.2, 6.9 ± 0.3, 12.3 ± 0.4, and 10.2 ± 0.3 GPa, respectively, and the Young’s modulus for the corresponding films was 95.2 ± 7.1, 115.1 ± 8.7, 131.7 ± 8.0, and 120.6 ± 7.6 GPa, respectively. The present *H* and *E* values were in a reasonable range compared with the previous studies [15,16,43]. Indeed, the of SnO_2_ film (F/Sn = 0) was comparable with the *H* = 6.1 ± 0.1 GPa and *E* = 78.5 ± 0.4 GPa of SnO_2_ films prepared by spray-pyrolysis [43]. Meanwhile, the *H* of 6.9–10.2 GPa and *E* of 115.1–131.7 GPa for the present FTO films were larger than *H* = 5.1 GPa and *E* = 71.1 GPa for FTO thin films deposited by chemical vapor deposition [16], and comparable with the *H* = 9.01 GPa and *E* = 125.24 GPa for the as-deposited FTO film prepared by APCVD [15].

**Figure 8:**
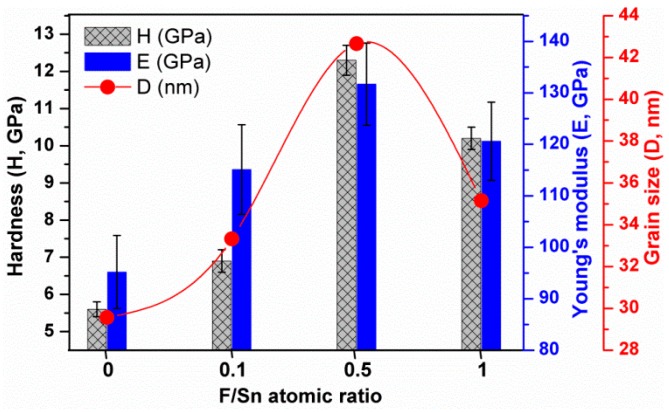
Hardness, Young’s modulus, and grain size of the FTO thin films prepared at various F/Sns from 0 to 1.

Figure 8 presents the F/Sn ratio dependence of the *H*, *E,* and *D* of the FTO thin films. The *H* and *E* results are mostly determined by the *D* value of FTO thin films, and follow an inverse Hall-Petch effect. A larger *D* leads to the larger *H* and *E* values. For examples, when *D* increased from 29.6 to 42.7 nm and then decreased to 35.1 nm, the corresponding *H* increased from 5.6 ± 0.2 to 12.3 ± 0.4 GPa and then decreased to 10.2 ± 0.3 GPa. It is worthy to note that the inverse Hall-Petch effect occurs in nanoscale granular materials with grain size below a certain critical value, for example 10 nm [44], and it is 42.7 nm for the FTO films in this study. In the inverse Hall-Petch effect, the film hardness is dominated by grain boundary sliding [15,45,46]. Regarding this mechanism, an increase of grain size will reduce the number of grain boundaries that in turn suppress grain boundary sliding, and lead to the nanomechanical hardening of the films. This *H*, *E* vs. *D* behavior agrees well with that of FTO thin films grown by APCVD [15]. In addition to the grain size, the internal porosity of polycrystalline materials influences the nanomechanical properties that *H* and *E* values decrease when increasing the level of porosity of the materials [47]. For the SnO_2_ film (F/Sn = 0), the surface morphology with spear-like particles may have internal voided structure near the surface due to the shadowing effect during the thin film growth [48,49]. This is partially attributed to the lower *H* and *E* values for the SnO_2_ film compared to those of FTO films. 

The plasticity index ($H/E_{f}$) and the plastic deformation resistance ($H^{3}/E_{f}^{2}$) of the FTO thin films were further calculated and reported in Table 2. The $H/E_{f}$ ratio characterizes the resistance of the material to elastic deformation, and a higher $H/E_{f}$ material is expected to have better wear resistant property due to small accumulated strain energy [50]. Meanwhile, $H^{3}/E_{f}^{2}$ describes the ability of a material in resisting plastic deformation and thus characterizes its toughness and resistance to crack propagation [29]. The $H/E_{f}$ values of the FTO thin films are in the range of 0.059–0.093 (Table 2). These $H/E_{f}$ values are larger than those of the Bi_3_Se_2_Te films (i.e., 0.028–0.031) [29], and reasonably smaller than the *H/E* > 0.1 values of some brittle materials, such as ceramic Mo-incorporation *β*-Ga_2_O_3_ films [51] and hard nanocomposite coatings with columnar microstructures [52]. In addition, the $H^{3}/E_{f}^{2}$ values increased from 0.019 to 0.107 GPa and then decreased to 0.073 GPa due to the variation of the grain size (Table 2). The FTO thin films prepared at F/Sn = 0.5 possessed the largest values of *H/E_f_* = 0.093 and $H^{3}/E_{f}^{2}$ = 0.107 GPa. This demonstrates that significant deformation resistance or toughness can be achieved in the FTO film with high crystallinity, large grain size, and dense morphology (i.e., F/Sn = 0.5). 

## 4. Conclusions

FTO thin films were successfully grown on glass substrates using USP. Effects of F/Sn atomic ratio on structural, morphological, compositional, electrical, optical, and nanomechanical properties of the films were studied. The films exhibited tetragonal rutile structure of SnO_2_ with dominant preferred orientation of (110), and the grain size tended to increase as the F/Sn ratio increased. The films had granular surface morphologies with spear-like particles for F/Sn = 0 and coconut shell-like particles for F/Sn = 0.1, 0.5, and 1.0, and they were compact and possessed good continuity. XPS results confirmed the presence of F-doping with concentrations of few percent (i.e., 0.7% for F/Sn = 0.1 and 5.1% for F/Sn = 0.5). By increasing F/Sn ratios, the films achieved significant reduction in resistivity from 4.1 mΩcm at F/Sn = 0 to 0.7 mΩcm at F/Sn = 1, primarily due to an order increase of *n* for F/Sn = 0.5 and 1.0. The films achieved excellent optical property, with an average transmittance in the visible range over 90% for F/Sn of 0–0.5. Importantly, the *H* and *E* of the FTO films were remarkably enhanced by introducing F-doping, following the inverse Hall-Petch effect. The highest *H* and *E* values were 12.3 ± 0.4 GPa and 131.7 ± 8.0 GPa for the FTO films prepared at F/Sn of 0.5 (with 5.1% F and *R_a_* of 11.9 nm). The FTO thin films prepared at F/Sn = 0.5 also possessed the largest plasticity index (*H/E_f_* = 0.093) and plastic deformation resistance ($H^{3}/E_{f}^{2}\;$ = 0.107 GPa). 

## Figures and Tables

**Table 1:** Preparation technique, percentage of F-doping, resistivity (*ρ*), carrier mobility (*μ*), concentration (*n*), mean free path (*L*), grain size (*D*), and transmittance at *λ* = 550 nm of the optimal FTO films in this study as compared to properties of the FTO films reported in the literature.

Sample	Preparation Technique	% F Dopant	*ρ* (mΩcm)	*μ* (cm^2^/Vs)	*n* ×10^20^ cm^−^^3^	*L* (nm)	*D* (nm)	*T* (%) (*λ* = 550 nm)	Ref.
FTO	USP	5.1	1.0	4.5	13	1	42.7	86.5	This study
FTO	USP	-	0.7	7.7	11.6	1.7	35.1	86.9	This study
FTO	USP	2.5	0.6	33.5	3.1	-	35	~68	[17]
FTO	USP	1	6.5	-	-	-	-	-	[19]
FTO	USP	5	1.6	4.8	8.4			68	[13]
FTO	SPD	7.5	1.5	21.9	1.9	2.5	25~33	-	[18]
FTO	APCVD	~4.8	0.53	23.8	5.0	3.8	20.1	84	[3]

**Table 2:** Grain size (*D*), hardness (*H*), Young’s modulus (*E_f_*), plasticity index ($H/E_{f}$), and plastic deformation resistance ($H^{3}/E_{f}^{2}$) of the FTO thin films prepared at various F/Sn atomic ratios.

F/Sn	D (nm)	*H* (GPa)	*E_f_* (GPa)	*H/E_f_*	$H^{3}/E_{f}^{2}$ (GPa)
0	29.6	5.6 ± 0.2	95.2 ± 7.1	0.059	0.019
0.1	33.3	6.9 ± 0.3	115.1 ± 8.7	0.060	0.025
0.5	42.7	12.3 ± 0.4	131.7 ± 8.0	0.093	0.107
1	35.1	10.2 ± 0.3	120.6 ± 7.6	0.085	0.073
